# Measuring patient satisfaction with education using extended reality in orthognathic surgery: Development, validation, and reliability of the 3D orthognathic surgery questionnaire (3DOS-Q)^☆^

**DOI:** 10.1016/j.pecinn.2025.100429

**Published:** 2025-08-29

**Authors:** Sander J.C. Tabernée Heijtmeijer, Noa Nicolai, Nard G. Janssen, Joël Kortes, Rick J.J. Pinkster, Behrus Puladi, Ashkan Rashad, O. Vladu, Peter A.J. Pijpker, Max J.H. Witjes, Sarina E.C. Pichardo, Joep Kraeima

**Affiliations:** a Department of Oral and Maxillofacial Surgery, University Medical Center Groningen, University of Groningen, Groningen, the Netherlands; b 3D Lab, University Medical Center Groningen, University of Groningen, Groningen, the Netherlands; c Department of Oral and Maxillofacial Surgery, University Medical Center Utrecht, Utrecht, the Netherlands; d Department of Traumasurgery, University Medical Center Groningen, Groningen, the Netherlands; e Department of Oral and Maxillofacial Surgery, University Hospital RWTH Aachen, Aachen, Germany; f Institute of Medical Informatics, University Hopsital RWTH Aachen, Aachen, Germany

**Keywords:** 3D models, Orthognathic surgery, Questionnaire, Education, Satisfaction, Validation, Virtual surgical planning

## Abstract

**Objectives:**

The effectiveness of extended reality technologies in improving patient understanding and satisfaction, particularly in orthognathic surgery, remains underexplored. No existing questionnaire specifically assesses patient satisfaction with different 3D model display techniques in this context. Therefore, this research aimed at developing and validating a questionnaire to assess patient satisfaction with preoperative education using 3D models for orthognathic surgery.

**Methods:**

Building on a previous questionnaire for trauma patients, an initial questionnaire was developed and adapted for orthognathic surgery patients based on literature and pretesting with patients and specialists (Phase I). The preliminary questionnaire underwent a field psychometric performance test in a randomized controlled trial at three centers specialized in orthognathic surgery (Phase II).

**Results:**

Nine patients and six specialists were included (Phase I), which resulted to a preliminary questionnaire consisting of twelve items. A total of 65 patients completed the preliminary questionnaire (Phase II). The final eleven items demonstrated strong psychometric properties, measuring a single distinct factor 4.99, Cronbach's alpha value of 0.87, for the German (α = 0.849) and Dutch (α = 0.836) versions. A Feldt test revealed no significant difference in internal consistency between the two languages (*p* = 0.401). The items together statistically significantly predicted patient satisfaction (*p* < 0.001).

**Conclusion:**

The final questionnaire has a good validity, total explained variance at 45.5 %.

**Innovation:**

The 3DOS-Q demonstrates significant potential as a tool for evaluating patient satisfaction with 3D models, suitable for extended reality technologies, improving patient care, and exploring satisfaction differences across patient groups and display techniques.

## Highlights

• No validated tool exists for patient education with 3D models in extended reality.
• The 3DOS-Q measures patient satisfaction as a single factor, PCA eigenvalue 4.99.
• 3DOS-Q shows strong internal consistency, Cronbach's α of 0.87.
• Validated in German and Dutch, with 45.5 % total explained variance in satisfaction.
• The 3DOS-Q effectively evaluates patient satisfaction suitable for extended reality.

## 1 Introduction

Informed consent is an important pillar of ethical medical practice, especially in the context of surgery where patients face major decisions about their health and well-being. It is crucial that patients fully understand the nature, risks and benefits of proposed treatments, especially in an elective procedure, so that they can make informed choices. [1] Effective communication between patients and their treating surgeons is essential to eliminate uncertainties and ensure that patients' preferences and values are taken into account, communication is also associated with improved health. [2] Shared decision-making is a central part of this communication and promotes a more patient-centered approach to care. [3,4]

This is especially relevant in orthognathic surgery (OS), which involves both functional and aesthetic considerations. [5] It is common practice to utilize 3D planning, or virtual surgical planning, in this field. This approach provides access to detailed 3D models of the patient planned outcome, enhancing both the surgical planning process and the ability to effectively educate patients about their treatment options and expected outcomes. Based on the patient's occlusion class —Class I (neutral), Class II (overbite), or Class III (underbite)— surgical interventions may involve the upper jaw with a Le Fort I osteotomy (LF1), the lower jaw with a bilateral sagittal split osteotomy (BSSO), or both jaws with a bimaxillary osteotomy (BIMAX).Unlike many other surgical procedures, OS is elective and addresses complex issues of jaw alignment and facial appearance, making it imperative that patients understand not only the technical aspects of the procedure, but also the potential impact on their quality of life and appearance. Informed consent in OS is therefore not only about understanding the medical details, but also about dealing with the subjective aspects of aesthetic outcomes. [6,7] Shared decision-making plays a crucial role for OS patients, as they are often given the opportunity to actively participate in their treatment plan. For instance, they may be able to choose whether they prefer a chin correction (genioplasty), surgery on both jaws, or just a single jaw procedure.

With the growth of the internet, patients now have unprecedented access to information about their health conditions and treatments. Traditionally, doctors were the primary source of information, and patients largely depended on their expertise. [8] However, patients today frequently search for additional details about their symptoms, diagnoses, and treatments, turning to online platforms for a more comprehensive view. While technologies like large language models have made it easier to access medical knowledge, they also increase the potential for misinformation, which can create confusion and alter patients' understanding and expectations of their care. [9]

Extended reality (XR) devices, such as virtual and augmented reality technologies, offer promising tools for improving patient education through immersive, interactive representations of surgical procedures and outcomes. [10] The 3D depiction of human anatomy can make it easier to explain diagnosis and treatments to patients. [11] XR devices are becoming increasingly readily available and accessible for health care providers. [12] Therefore the use of XR is growing, however, despite their potential, current literature on the effectiveness of XR devices in improving patient understanding and satisfaction is limited. [13,14] Moreover, there is currently no questionnaire available that is specifically designed to assess orthognathic patients' satisfaction with various 3D model display techniques. Such a tool is essential to determine which methods lead to greater patient satisfaction and understanding of their jaw abnormality and proposed treatment, which is crucial for improving shared decision-making and overall patient satisfaction. Moreover, the impact of XR technologies can vary significantly depending on the cultural context and team practices in different regions. [13] Understanding these factors is essential for effective patient management and is vital for effectively integrating XR devices into patient education strategies and ensuring that they meet the diverse needs of patients. [14] Therefore, the aim of this study was to develop and validate a questionnaire specifically designed to measure patient satisfaction with preoperative education in OS using 3D models, whether displayed on a standard computer screen or through XR technology.

## 2 Methods

This study was designed as a two-stage process, consisting of questionnaire development (phase one) and clinical validation in a multi center randomized controlled trial (RCT) (phase two).

### 2.1 Phase 1 – Questionnaire development

In the first phase of the study, the objective was to develop an initial questionnaire to measure patient satisfaction and to gather evidence for *content validity*. This process was informed by our previous reported questionnaire [15] on educating patients with fractures in the department of trauma surgery with 3D models, existing literature on language use in OS, and pretesting among a group of OS patients and medical specialists specialized on OS patients. The original questionnaire was validated for Dutch-speaking patients and measured patient satisfaction and two sublevels: clarity of the images and importance of the images for the patient. The original questionnaire was adapted to be more suitable for OS patients.

Nine patients were divided into two groups: one group viewed 3D models on a computer monitor, while the other group viewed 3D holographic images. Six surgeons were shown the holographic models as they were already accustomed to 3D models on computer screens. The qualitative Three-Step-Test-Interview method [16] was employed to assess the clarity, relevance, and completeness of the questionnaire. This approach provided data on the participants' response behavior and opinions on the questionnaire were gathered.

Thematic analysis was performed using Braun and Clarke's [17] six-phase approach to analyze the interview data. Data from both groups contained information relating to the subjects' overall impressions of the questionnaire, formulation, content, format, and attitudes towards medical imaging. The patient and specialist data were analyzed separately [17] to compare their perspectives. This helped identify any conflicting views and assess which opinions should be prioritized to determine the questionnaire's suitability for OGS patients. To enhance inter-rater reliability, an independent psychology student with prior knowledge and experience in the process of qualitative coding reviewed a random transcript from each group using the finalized coding scheme. This process aimed to improve intercoder agreement and ensure consistent interpretation of codes. Based on the collected opinions and in the light of existing data a preliminary questionnaire was developed, which is validated in Phase II.

### 2.2 Phase 2 – Clinical validation

#### 2.2.1 Study design

In a multi-center study, three academic medical centers in the north-western region of Europe, participated. UMC Groningen (Center A), UMC Utrecht (Center B) and RWTH Aachen (Center C). The preliminary version of the 3DOS-Q was clinically tested to assess the psychometric properties in patients scheduled for OS during their intake consultation. Patients were consulted and asked to complete the preliminary version in their native language, Dutch or German. Sixty patients (20 per center) were included for this study from August 2023 to June 2024. After the intake consultation referred patients for OS were asked by the treating physician if they wanted to participate in this study. Patients were randomly assigned to one of two groups according to a random allocation rule: (1) Monitor group (MON) and (2) Augmented reality group (AR).

#### 2.2.2 3D consultation process

**Fig. 1:**
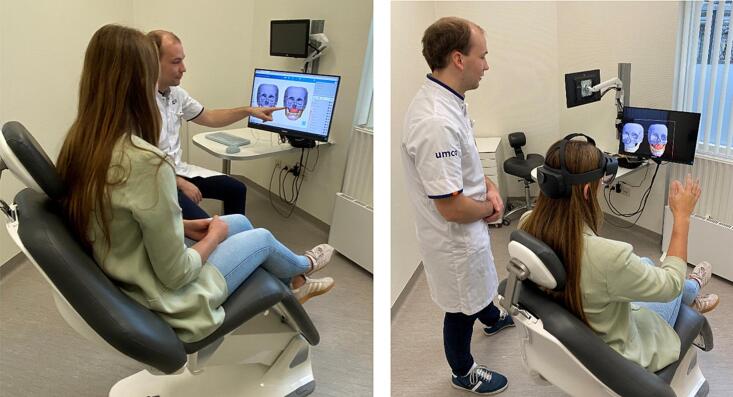
Example of education for both groups. Two images of patient education with 3D models on a monitor (left) and 3D models viewed with an augmented reality device on the head (right). In the left image, the monitor group, the patient watches the models on a monitor while receiving explanations from the researcher. In the right image, the explanation is performed by the researcher while the patient views the models via the HoloLens II and can interact with them. The researcher can see the patient's perspective on the monitor and give instructions for the software.

Upon agreement, after signed informed consent, the patient was taken to a separate examination room where the patient was educated with 3D medical models and the patients were informed about their jaw deviation and proposed treatment according to a standardized protocol. The procedure was explained using 3D models of a demonstration patient corresponding to the proposed treatment plan and occlusion class of the patient. The models illustrated the surgical process and visualized the jaw position before and after surgery and the effect of surgery on the aesthetics of the patient. The techniques used to visualize the 3D models differed between the groups: In the MON group, the models were presented in 3D via a monitor trough the Mimics Viewer application (Materialise, Leuven, Belgium) while in the other group, the patient wore an AR Head Mounted device (HMD) (HoloLens 2, Microsoft) that presented the same 3D models as holograms floating in the room using the Mimics Viewer XR application (Materialise, Leuven, Belgium). The groups can be seen in Fig. 1. There was a time-limit of 20 minutes for the 3D consultation to prevent a difference in spent time and therefore a bias between the two groups. At the end of the 3D consultation the preliminary 3DOS-Q was provided to the patient in their native language to evaluate their satisfaction on the education with 3D models.

#### 2.2.3 Statistical analysis

Analyses were conducted using SPSS (version 28, IBM, Chicago, IL, USA). The analysis process involved five key steps to select the final items and evaluate the psychometric properties of the 3DOS-Q.

• First, Inter-item correlations and item response distributions were calculated. Items with correlations above 0.7 were considered potentially redundant. [17] One item from each pair was considered to be removed based on item wording and content coverage. Floor and ceiling effects were assessed by examining response distributions. Items where over 80 % of respondents selected either the lowest or highest response option were deemed inappropriate and excluded.
• Principal component analysis (PCA) was to determine the amount of factors behind the items remaining and to determine whether the questionnaire should be rated as a total scale or as individual subscales. Sample size requirements for PCA are commonly estimated using a rule of thumb of 5 to 10 participants per item. [18] For the 12-item questionnaire, a minimum of 60 participants was therefore targeted to ensure an adequate basis for factor analysis. The Kaiser-Meyer-Olkin (KMO) measure of sample adequacy was used, with a minimum KMO of 0.5 required for factor analysis. [19]
  To determine the number of underlying factors, a parallel analysis was conducted based on 100 randomly generated datasets, matched to the structure of the actual data (65 participants, 12 items), following the procedure described by O'Connor [20]. For each simulated dataset, a PCA was conducted, and the 95th percentile of eigenvalues was extracted. Factors in the actual data were considered significant only if their eigenvalue exceeded the 95th percentile of the corresponding simulated eigenvalues.
  An initial PCA with varimax rotation was conducted without constraining the number of factors. Subsequently, fixed-factor PCAs were performed, based on both the structure of the original questionnaire (which contained two factors [15]) and the outcome of the parallel analysis. In all PCAs, items were evaluated based on the following predefined criteria:

(1) A minimum factor loading of ≥0.40 on at least one component;
(2) No substantial cross-loading, defined as a difference < 0.10 between the two highest loadings;
(3) Conceptual alignment with the underlying construct.

Items that failed to meet these criteria were considered for removal in an iterative process, in which PCA was repeated after each item exclusion to assess the impact on the factor structure. This stepwise procedure continued until all retained items met the inclusion thresholds. The decision to adopt a model was made based on the results of the parallel analysis and the interpretability of the resulting components. [21]

• The internal consistency of the total scale or subscales resulting from the PCA was evaluated using Cronbach's alpha (α). A minimum Cronbach's α of 0.7 was considered necessary for sufficient internal consistency for analysis of scale scores at the group level. [21] If this criterion was not met, removal of additional items was considered to improve α.
• To evaluate the discriminant validity of the factor, Spearman's ρ correlations were calculated for the total score and, where applicable, for the subscales. To ensure sufficient independence, subscales were expected to exhibit at most moderate correlations (ρ < 0.70), indicating that they represent distinct factors of patient satisfaction with imaging. Additionally, multiple linear regression analysis was conducted to determine whether the items and subscales were significantly and independently associated with the global satisfaction rating for 3D medical images.
• Since the previously reported questionnaire was assessed and validated only in Dutch and for native Dutch speakers, the developed preliminary questionnaire was also translated to German according to the forward-backward principle. [22] German specialists were asked to evaluate the German version on suitability. In addition, additional analyses were conducted to evaluate the validity of the questionnaire in German.

## 3 Results

### 3.1 Phase I – Questionnaire development

The evaluation process revealed that while the nine patients and six surgeons largely agreed on the clarity of the original questionnaire's language, they differed regarding its applicability to OS patients. The evaluation revealed that the previous reported questionnaire [15] was partially effective for measuring satisfaction with medical images in OS patients. This partial effectiveness was attributed to the focus of the original questionnaire on a different patient population, its timing of implementation, the use of models of another demo patient and the imaging techniques used. Insights gained from this evaluation, along with existing data, informed the necessary adaptations to the questionnaire. As part of establishing content validity, Terminology was refined to be more relevant and comprehensible for OS patients, with certain terms specified or explained in simpler language. Redundant or unnecessary questions were removed, and inapplicable items were either excluded or modified to better suit the needs of OS patients. Additionally, several questions were rewritten to be more concise, ensuring clarity and ease of use. The translation to German produced a German version of the questionnaire, which was translated back to the source language to eliminate discrepancies. The translation was revised and reassessed with the translation institution and specialists at OS with Dutch and German as native languages, which resulted in a preliminary version in German too.

According to the result of phase I, the preliminary 3DOS-Q contained three questions about demographics, 12 Likert-type satisfaction questions, one 1–10 numerical rating scale for overall general satisfaction, a yes/no question on whether to recommend the use of images, and an open text field for respondents to explain their answers in more detail or provide additional comments. Demographic questions included sex, age and most recent level of education. The response scale for the 12 Likert-type items ranged from completely disagree (1) to completely agree (5). The overall rating scale of 1–10 asked patients about their overall satisfaction with the use of 3D images, with 1 indicating “very dissatisfied” and 10 indicating “very satisfied. The final question asked whether respondents would recommend the use of 3D images when consulting other patients. This question could be answered yes or no, while respondents also had the opportunity to further explain their answer or add additional information if desired. Table 1 shows the preliminary version, which was then validated in the subsequent phase.

**Table 1:** Preliminary questionnaire, the items have been translated into English from the original German and Dutch questionnaire.

Items
1. The 3D medical images provided clear information about my abnormal jaw position
2. The 3D medical images were necessary to understand the explanation of my jaw deviation and surgery.
3. Seeing the 3D medical images is going to help me make a conscious decision with the medical specialist about my treatment.
4. I understood the explanations given when showing the 3D medical images.
5. The 3D medical images motivate me to enter the treatment process.
6. I expected to see medical images during my consultation
7. Seeing 3D medical images of my deviated jaw position was an important part of the appointment for me.
8. The 3D medical images made me better understand the explanations during my appointment.
9. Because of the 3D medical images, I understand my own jaw deviation better.
10. Because of the 3D medical images, I can better imagine how my surgery will go.
11. The 3D medical images are suitable for explaining my jaw deviation and the course of the operation.
12. The 3D medical images were reassuring for me to see during the appointment.

### 3.2 Phase II – Clinical validation

#### 3.2.1 Participants

In total 68 patients participated in this study. Two patients did not fill in an overall rating score, one patient did not fill in the last Likert-scale question, therefore these patients were excluded in the analyses. One participant was excluded due to systematic inconsistent responses (all items on the Likert-scale scored “totally disagree” despite positive overall ratings). Sensitivity analysis confirmed this exclusion did not affect conclusions. Therefore, the exclusion was retained in the final analysis. Center A included 25 patients, Center B included 20, and Center C included 20. 33 Patients completed the questionnaire after consultation with 3D models on a monitor, 32 patients with 3D models seen in AR. The mean age of the patients was 28 years (SD 12.2, range: 16–59), 39 (60.0 %) patients identified as women, 26 (40.0 %) patients identified as men. Most patients had a Class II occlusion 46 (70.8 %), 17 patients had a Class III occlusion (26.2 %), and two patients had a Class I occlusion (3.1 %). Patients were well distributed across the different education levels which were from primary school only (Level 1) to scientific education (Level 7), with the exception of Level 0. Twenty patients were native German speakers, and forty-five patients were native Dutch speakers. Each group completed the questionnaire in their respective native language. Table 2 shows an overview of the patient characteristics.

**Table 2:** Overview of the characteristics of the included patients.

Patient characteristics	Total	Dutch speakers (*N* = 45)	German speakers (*N* = 20)
Education Modality, n (%)			
Monitor	33 (50.8)	23 (51.1)	10 (50.0)
Augmented Reality	32 (49.2)	22 (48.9)	10 (50.0)
			
Age, mean (SD [range])	28.35 years (±12.20 [16–59])	29.31 years (± 13.04 [16–59])	26.20 years (± 10.05 [18–57])

Gender, n (%)			
Men	26 (40.0)	28 (62.2)	11 (55.0)
Women	39 (60.0)	17 (37.8)	9 (45.0)

Occlusion Class, n (%)			
Class I	2 (3.1)	2 (4.4)	0 (0.0)
Class II	46 (70.8)	37 (82.2)	9 (45.0)
Class III	17 (26.2)	6 (13.3)	11 (55.0)

Treatment, n (%)			
Solitary LF1^1^	4 (6.2)	3 (6.7)	1 (5.0)
Solitary BSSO^2^	24 (36.9)	22 (48.9)	2 (10.0)
BIMAX^3^	37 (56.9)	20 (44.4)	17 (85.0)

Education Level, n (%)			
1 - Primary School	0 (0.00)	0 (0.0)	0 (0.0)
2 - Lower Vocational Education	6 (9.23)	6 (13.3)	0 (0.0)
3 - Intermediate Vocational Education	18 (27.69)	16 (35.6)	2 (10.0)
4 - Senior General Secondary Education	5 (7.69)	0 (0.0)	5 (25.0)
5 - Pre-University Education	11 (16.92)	4 (8.9)	7 (35.0)
6 - Higher Professional Education	13 (20.00)	12 (26.7)	1 (5.0)
7 - Scientific Education	12 (18.46)	7 (15.6)	5 (25.0)

1 LF1, an osteotomy of the maxilla to reposition the upper jaw.

2 BSSO, an osteotomy of the mandible to reposition the lower jaw.

3 BIMAX, an osteotomy of both jaws, consisting of a BSSO and a LF1.

To assess differences between the German- and Dutch-speaking groups, Mann–Whitney *U* tests (for age) and Chi-square tests (for gender and treatment group) were performed. No significant differences were found in age (Mann–Whitney U, *p* = 0.989), gender (χ^2^, *p* = 0.583) or treatment group allocation (χ^2^, *p* = 0.934).

For variables such as occlusion class, treatment type (e.g., single jaw vs. bimaxillary surgery), and educational level, the number of participants per category was too small to perform valid statistical testing. However, based on the raw frequencies, the German-speaking group appeared to include more patients with Class III malocclusion and a higher proportion of bimaxillary procedures.

#### 3.2.2 Item quality

Item analysis indicated one item with low correlation to all other questions: item 6: (“I expected to see medical images during my appointment.”). Items 10 (“The 3D medical images allow me to better imagine how my surgery will go”) & 11 (“The 3D medical images are suitable to explain my jaw position and the process during surgery”) had a high inter-item correlation of 0.73. Despite the high correlation, both items were kept for further structural validity analysis, because the formulation and content coverage does differ between the two questions. The response frequencies of the items showed that no item was scored by more than 80 % of the respondents in the lowest or highest response category, which indicated the absence of a floor or ceiling effect.

#### 3.2.3 Structural validity

**Fig. 2:**
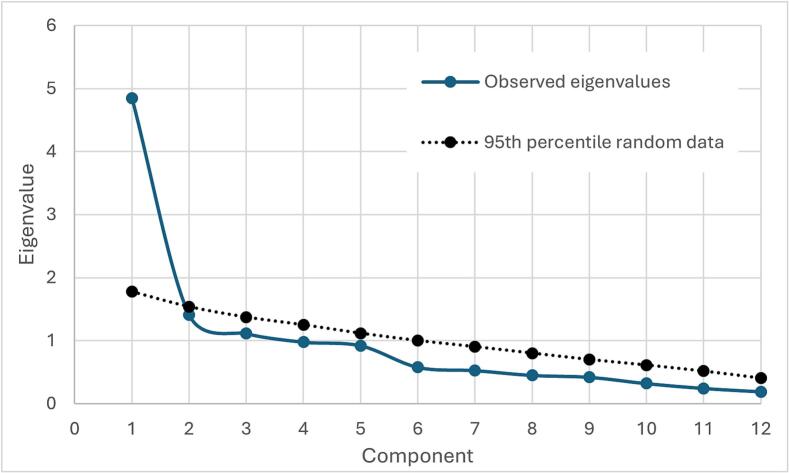
Scree plot of observed eigenvalues and 95th percentiles of eigenvalues from parallel analysis of 100 random datasets.

Ultimately, 65 participants were included in the analysis, which exceeded the minimum required sample size for PCA and thus supports the robustness of the factor analytic approach. The KMO value of 0.83 indicated that the data was suitable for executing PCA. Three different factors with eigenvalues >1 were found in the initial PCA, which explained 61.42 % of the variance. However, item 8 (“The 3D medical images help me better understand the explanations during my appointment”) had a positive factor loading of 0.55 for two components. Consequently, PCAs were performed with a fixed number of two factors, as the previous questionnaire consisted of two factors, resulting in items 8 with a difference in factor loading less than 0.1 and item 9 with two high factor loadings of 0.65 and 0.48. Parallel analysis indicated that only the eigenvalue of the first factor exceeded the 95th percentile of the distribution of eigenvalues derived from random data. The scree plot is shown in Fig. 2. Consequently, and due to the small factor differences between two items, only one fixed factor was used. The first factor had a high value of 4.85 and all items had a factor loading >0.4. Item 6 was excluded because it had a low correlation (Range − 0.190 – −0.027) with other questions and a negative factor loading (−0.188) in the one-factor PCA. The factor loadings can be seen in Table 3.

**Table 3:** Final factor structure and individual p-value for prediction of overall satisfaction score.

Items	Factor loadings	Satisfaction correlation (p-value)
1. The 3D medical images provided clear information about my abnormal jaw position	0.50	0.212
2. The 3D medical images were necessary to understand the explanation of my jaw deviation and surgery.	0.56	0.950
3. Seeing the 3D medical images is going to help me make a conscious decision with the medical specialist about my treatment.	0.60	0.012
4. I understood the explanations given when showing the 3D medical images.	0.60	0.021
5. The 3D medical images motivate me to enter the treatment process.	0.68	0.237
6. Seeing 3D medical images of my deviated jaw position was an important part of the appointment for me.	0.60	0.196
7. The 3D medical images made me better understand the explanations during my appointment.	0.79	0.033
8. Because of the 3D medical images, I understand my own jaw deviation better.	0.80	0.693
9. Because of the 3D medical images, I can better imagine how my surgery will go.	0.75	0.011
10. The 3D medical images are suitable for explaining my jaw deviation and the course of the operation.	0.75	0.142
11. The 3D medical images were reassuring for me to see during the appointment.	0.56	0.521
Eigenvalue	4.818	
Explained Variance	43.81 %	45.5 %

#### 3.2.4 Internal consistency

The final single factor structure resulting from the preceding analyses showed a high Cronbach's α value of 0.87. No items were removed during this analysis, because removal of items would result in a lower value.

#### 3.2.5 Discriminant validity

Previous analyses showed that the 3DOS-Q has 1 factor, therefore discriminant validity of underlying factors was not determined. A multiple regression was run to predict the overall patient satisfaction score based on the final eleven items. These items together statistically significantly predicted patient satisfaction, *F*(11, 52) = 9.426, *p* < 0.001, *R*^*2*^ = 0.666, *adj. R*^*2*^ = 0.595. Table 3 shows the *p*-value of the satisfaction correlation. Items 3,4,7, and 9 added statistically significantly to the prediction, *p* < 0.05. Analysis of the total score of global satisfaction confirmed the discriminant validity (β = 0.67, *p* ≤ 0.01). The total explained variance in global satisfaction was good at 45.5 %.

To assess the validity of the questionnaire in German, Cronbach's alpha was calculated for the German (α = 0.849) and Dutch (α = 0.836) versions of the questionnaire. A Feldt test [23] revealed no significant difference in internal consistency between the two languages (p = 0.401). Additionally, Mann-Whitney U tests showed that patients who completed the German version reported significantly higher satisfaction than those who completed the Dutch version for the final 11 items (*p* < 0.001) and for the one 1–10 numerical rating scale for overall general satisfaction (*p* = 0.004).

## 4 Discussion and conclusion

### 4.1 Discussion

The aim of this article was to develop a validated questionnaire (Phase I) to determine patient satisfaction with their 3D consultation for OS within the oral and maxillofacial surgery department. The questionnaire was validated with a similar number of patients educated with 3D models on a screen and via XR technology. The final 3DOS-Q is a quick-to-use, reliable and valid measure of patient satisfaction with the use of medical 3D models for intake consultations (Phase II). It is easy for patients to complete because the burden is low, consisting of only 11 Likert-scale questions. The final eleven items have adequate item quality, measure one distinct factor and a high internal consistency. The scoring of the eleven items showed discriminant validity regarding overall satisfaction with 3D models for patient consultations. Overall, the 3DOS-Q is promising as an instrument to measure patients' satisfaction with 3D models in detail. It is well suited for improving patient care and investigating differences in satisfaction between patient groups and different 3D model display techniques.

Several studies have been conducted to assess the satisfaction of patients educated with 3D models and virtual surgical planning with or without an extended reality technique. [[24], [25], [26], [27], [28], [29]] However, to the best of our knowledge no study yet has been performed to develop a validated questionnaire for patient education with 3D models and XR technology in a multicenter setting. Previously performed studies on patient satisfaction used generic validated questionnaires or non-validated self-developed questionnaires, which are often very limited. The study of Colback et al. [29] can serve as an example of this problem, the unvalidated questionnaire to measure satisfaction consisted of only two questions, patients were asked which technique they would recommend and which helped the most. From this, it becomes clear that there is a real need for a validated questionnaire suitable for education with XR technology.

While the original questionnaire we developed, [15] measured patient satisfaction using two components, the analysis of the 3DOS-Q for orthognathic patients identified only one significant component. In the original questionnaire, the number of questions associated with the second component was already limited and differed significantly from those linked to the first component. Similarly, the preliminary version of the 3DOS-Q included eight questions about the importance of seeing the 3D medical models for the patient, compared to only four questions about the clarity of the 3D images. Additionally, the second component of the original questionnaire had an almost acceptable but lower Cronbach's alpha value. Therefore, it is not surprising that this analysis does not divide patient satisfaction into two subscales, as the original questionnaire did. The original questionnaire already recommended assessing the robustness of the two-factor structure, given the exploratory nature of the PCA. Precisely what this study shows is that this structure does not hold with a different patient group. Importantly, the absence of distinct subcategories does not diminish the clinical utility of the questionnaire: the 3DOS-Q remains a valid and practical tool to assess overall patient satisfaction with the use of 3D images in preoperative communication. We interpret the single factor as representing a broad construct of patient satisfaction that integrates perceived clarity, relevance, and emotional impact of the 3D images. This suggests that for orthognathic patients, satisfaction with the imaging is experienced as a unified construct rather than as separable components. Consequently, the questionnaire is best used as a measure of overall satisfaction rather than for assessing distinct subdomains.

The results of the validity of the questionnaire in German showed good internal consistency, the small difference in the Alpha scores between the two languages suggests that the questionnaire is equally reliable in both German and Dutch. However, there was a significant difference in satisfaction between the group that had completed in German and the group that had completed in Dutch. The German version of the questionnaire was carefully developed using a forward–backward translation procedure and reviewed by German specialists to ensure conceptual accuracy and contextual suitability. All centers followed an identical protocol for patient education, using identical 3D images and providing information to similar patient populations. The order, setting, and conditions for completing the questionnaire were standardized. However, data collection was conducted by different researchers in each country, and the sample sizes varied (Germany: *n* = 20; The Netherlands: *n* = 45). These factors may have introduced bias. While methodological differences cannot be ruled out as a contributing factor, cultural influences may also have played a role For instance, In the more hierarchical healthcare structure in Germany, physicians are perceived to have a higher level of expertise, which could lead to higher satisfaction in patients. [30] This is consistent with previous findings where German patients reported higher satisfaction compared to two other countries even though the patients had poorer outcomes. [31] It is also important to remember that satisfaction measurements are presumed to have low discriminative ability, meaning they may not effectively demonstrate differences between hospitals. Therefore, it would be more appropriate to examine the differences within a single center. [30] Overall, the observed difference in satisfaction likely reflects a combination of methodological variation and cultural context, and should be interpreted with caution.

One limitation of this study is that the total explained variance in global satisfaction was modest at 45.9 %, suggesting that patients' satisfaction with 3D models is influenced by factors beyond the importance and clarity of the imaging. This suggests that significant unmeasured factors likely contribute to patient satisfaction and are not captured by the current questionnaire. While this restricts the proportion of variance explained, it also emphasizes important areas for future research to identify and include these determinants in satisfaction measures. This relatively low explained variance may be attributed to two main factors. First, the questionnaire might not capture all relevant aspects of satisfaction. As noted by Phelps et al. [11], satisfaction comprises seven categories, and compared to the original 22-item questionnaire, our current version may be missing important dimensions such as *Uncomfortableness* and *Vulnerability*. This was a deliberate trade-off in order to maintain the instrument's conciseness, practicality, and feasibility for clinical use. Second, environmental factors could also significantly influence overall satisfaction but are not fully captured by the questionnaire. Compared to the original questionnaire [15], the total explained variance is higher. However, It is currently unclear what factors might impact satisfaction, but one possibility is that it is shaped by stable personality traits of the patients. [32] Denadai et al. found that satisfaction with facial appearance overall for OS patients was significantly correlated to psychological well-being. [33] Further research is needed to test this hypothesis and to identify other variables that predict satisfaction with imaging. Another possible limitation is that the researcher was present in the same room while the patients were completing the 3DOS-Q. Although the researcher could not and did not directly observe the answers of the patients, this presence may have led to socially preferable answers. [34] However, the decision to be present was made to ensure a high response rate and to be able to answer any questions from patients immediately. [35] Four items added statistically significant to the prediction of global satisfaction but seven items did not. This finding suggests that, although the significant items are strong direct predictors of satisfaction, the remaining items still showed good factor loadings despite their non-significant individual correlations. This indicates that they are part of a shared underlying construct. Item 2 (*p* = 0.95), for example, focuses on the necessity of 3D images for understanding the diagnosis and treatment plan. Although it had a high factor loading, it did not predict satisfaction. This finding supports the hypothesis that the 3DOS-Q questionnaire may capture a construct broader than satisfaction alone. Although the 3DOS-Q was designed to measure patient satisfaction, the single underlying factor appears to capture a broader concept. This construct includes patients' perceived understanding of their condition, the informational value of the 3D images, the effectiveness of communication, and their sense of involvement in decision-making. Highlighting this broader scope clarifies that the questionnaire reflects the overall educational and communicative impact of the 3D consultation, not just subjective satisfaction. We propose that this latent factor reflects concepts such as the perceived informational value of the images, the effectiveness of health communication, and informed decision-making. These dimensions could influence how patients process and evaluate the use of 3D images independently of their level of satisfaction. Future research could explore and validate this underlying construct through confirmatory factor analysis or by integrating qualitative measures to better understand patient interpretation.

Understanding has already been mentioned by Phelps et al. [11] as an important factor. While the 3DOS-Q includes items related to patients' subjective understanding of their jaw condition and treatment, the relationship between 3DOS-Q scores and objective measures of patient knowledge remains to be established. Future studies should investigate whether scores on the 3DOS-Q are correlated with patients' objective knowledge of their condition, to gain further insight into the broader scope of the instrument's validity and its potential to reflect not only subjective perceptions but also informed patient understanding.

The questionnaire was evaluated only in its Dutch and German versions, which limits its applicability to native speakers of these languages. Future studies should aim to translate, adapt, and validate the questionnaire for speakers of other languages to enhance its usability and reliability across diverse clinical settings. This process would help ensure that the translated items are culturally and linguistically appropriate, retain their psychometric properties, and remain effective in measuring patient satisfaction across different language groups. In this study, the global satisfaction rating scale was used to assess the validity of the single factor underlying the 3DOS-Q. An alternative approach for refining the 3DOS-Q could involve selecting individual items that most strongly predict global satisfaction (e.g., through regression analysis) or another primary metric. Future studies could use such a method to further shorten the 3DOS-Q while enhancing its predictive power. One concern, which we believe also applies to the validated questionnaire presented, is that it was designed specifically for orthognathic patients. In the future, it would be valuable to develop a questionnaire that could effectively measure satisfaction with XR technology education for a wider range of patient groups. Although an attempt was made to generalize the questionnaire during development, terms familiar to patients, such as ‘jaw deviation’, were used to address specific medical issues, ensuring clarity and relevance. However, this limited its wider applicability. The solution to this problem has not yet been identified and should be addressed in future research to ensure more versatile applicability in different patient populations. Importantly, this limitation concerns the questionnaire itself rather than the methodological framework. The approach adopted here provides a transferable model for developing and validating patient satisfaction measures for other surgical and medical populations.

### 4.2 Innovation

Informed consent and patient education are crucial for surgical procedures, especially in OS, where both function and aesthetics are addressed. [5] The 3DOS-Q can be used in studies to determine which techniques for displaying 3D models, a monitor or XR technology, lead to greater patient satisfaction and better understanding of their jaw deviation and the proposed treatment. Shared decision-making plays a key role for OS patients as they frequently have the potential to actively participate in their treatment plan. By enhancing patient satisfaction as measured by the 3DOS-Q, shared decision-making can be significantly improved, which is essential for achieving overall patient satisfaction.

### 4.3 Conclusion

In conclusion, the 3DOS-Q is the first validated questionnaire specifically designed to assess OS patients satisfaction with 3D models used during clinical consultations. This study demonstrates that the 3DOS-Q, with its single-factor structure, is a reliable and valid instrument for this purpose. The 3DOS-Q offers valuable insights into patient satisfaction and can help enhance the effectiveness of 3D models in patient education before OS.

## Statements

All procedures performed in this study involving human participants were in accordance with the ethical standards of the institutional and/or national research committee(s) and with the 1964 Helsinki Declaration and its later amendments or comparable ethical standards. The Medical Ethical Committees of the three mentioned hospitals reviewed the study design and determined that full review and approval were not needed (Reference Number: 2023/524 METc Groningen).

Informed consent was obtained from all individual participants included in this study. The privacy rights of human subjects were observed throughout the research process.

## CRediT authorship contribution statement

**Sander J.C. Tabernée Heijtmeijer:** Writing – review & editing, Writing – original draft, Visualization, Software, Project administration, Methodology, Investigation, Formal analysis, Data curation, Conceptualization. **Noa Nicolai:** Writing – review & editing, Methodology, Investigation, Data curation, Conceptualization. **Nard G. Janssen:** Writing – review & editing, Supervision, Methodology, Investigation, Conceptualization. **Joël Kortes:** Writing – review & editing, Supervision, Methodology, Investigation, Conceptualization. **Rick J.J. Pinkster:** Writing – original draft, Methodology, Formal analysis. **Behrus Puladi:** Writing – review & editing, Investigation, Data curation. **Ashkan Rashad:** Writing – review & editing, Investigation, Data curation. **O. Vladu:** Writing – review & editing, Investigation, Data curation. **Peter A.J. Pijpker:** Writing – review & editing, Supervision, Methodology, Conceptualization. **Max J.H. Witjes:** Writing – review & editing, Supervision, Methodology, Investigation, Conceptualization. **Sarina E.C. Pichardo:** Writing – review & editing, Supervision, Methodology, Investigation, Conceptualization. **Joep Kraeima:** Writing – review & editing, Supervision, Methodology, Conceptualization.

## Declaration of competing interest

The authors declare the following financial interests/personal relationships which may be considered as potential competing interests:

Tabernée Heijtmeijer reports equipment, drugs, or supplies was provided by Materialise NV. If there are other authors, they declare that they have no known competing financial interests or personal relationships that could have appeared to influence the work reported in this paper.
